# Markers Associated with Starch, Protein and Asparagine Content in Grain of Common Wheat

**DOI:** 10.3390/genes16060661

**Published:** 2025-05-29

**Authors:** Kinga Rączka, Przemysław Matysik, Tadeusz Drzazga, Ada Dorczyk, Marta Olejniczak-Idczak, Dorota Tyrka, Mirosław Tyrka

**Affiliations:** 1 Doctoral School of the Rzeszów University of Technology, Powstańców Warszawy 6, 35-959 Rzeszów, Poland; d556@stud.prz.edu.pl; 2 Plant Breeding Strzelce Group IHAR Ltd., Główna 20, 99-307 Strzelce, Poland; p_matysik@hr-strzelce.pl (P.M.); m_idczak@hr-strzelce.pl (M.O.-I.); 3 Małopolska Plant Breeding Ltd., Sportowa 21, 55-040 Kobierzyce, Poland; tdrzazga@mhr.com.pl; 4 Plant Breeding Smolice Ltd., Smolice 146, 63-740 Kobylin, Poland; ada.dorczyk@hrsmolice.pl; 5 Department of Biotechnology and Bioinformatics, Rzeszow University of Technology, Powstańców Warszawy 6, 35-959 Rzeszów, Poland; dtyrka@prz.edu.pl

**Keywords:** association studies, silicoDArT, single nucleotide polymorphism

## Abstract

**Background:** Grain protein (GPC) and grain starch (GSC) content in common wheat determines suitability for further end-use processing and is an important quality factor. The level of free asparagine in grains (GFAC) significantly affects suitability for thermal processing. The aim of this genome-wide association study (GWAS) was to identify markers associated (MTA) with the levels of GPC, GSC and GFAC in elite winter wheat breeding lines, and to identify candidate genes. **Methods:** In total, 344 winter wheat lines were phenotyped and genotyped with DArTseq markers. **Results:** This GWAS revealed 14 MTAs for GPC, 40 for GSC and 43 for GFAC. The new markers were identified and explained from 6.3% to 12.2% of phenotypic variation. For GPC, the region adjacent to marker 4990459 (QGpc.rut.2D) explained 10.2% of the variation and was stable between two years. The novel gene TraesCS7A03G037500, encoding sucrose synthase involved in starch biosynthesis, was identified in the proximity of QGsc.rut.7A.2. The TraesCS1B03G0736700 gene, coding NAD(P)H dehydrogenase subunit H involved in the mitochondrial electron transport chain, was found in the proximity of QGfac.rut.1B.1. **Conclusions:** These findings provide valuable insights for elucidating inheritance of GCS, and the identified MTAs provide molecular markers for the reduction of free asparagine and increase of protein content in wheat grains.

## 1. Introduction

Common wheat (*Triticum aestivum* L.) grown worldwide on more than 213 million hectares is, beside rice and corn, the third most important crop [1]. It is expected that the demand for this crop will increase by about 70 percent over the next 30 years [2]. Therefore, wheat yield improvement is a very important worldwide issue. Almost 60% of produced wheat is destined for food [3], and the main storage components of wheat grains are starch and proteins [2,4]. The content of starch and proteins is different in segments of wheat grain. The main (80–85% of the dry weight) source of starch in wheat grain is endosperm consisted in 65–80% of starch. An embryo and bran contribute to 2–3% and 13–17% of dry weight, respectively, and contain below 2% of starch [5,6]. Proteins are more evenly distributed in the wheat embryo (20–25%), bran (10–15%) and endosperm (7–15%) [6,7,8]. In wheat, the most important quality yield are related to GSC and GPC. Starch and grain protein content are quantitative, interdependent traits, dependent on both genetic predisposition and environmental conditions [9,10] such as crop rotation, annual rainfall, cultivation practices, soil fertility and nitrogen fertilizer [10]. The control of these traits takes place through the synchronized action of a number of loci [10,11]. Wheat produces transitional and storage starch with the enzymes coded by waxy (Wx) genes [9,12,13,14,15]. Genetic variation in genes involved in starch synthesis and accumulation during grain development directly affects wheat yield and quality of grains [16,17]. Similarly, the total protein content, its composition and the gliadin–glutenin ratio affects the quality of the flour and the bakery products [8,9,10,18,19].

Identification and utilization of loci responsible for GSC and GPC along with appropriate crop management appears to be a suitable agricultural strategy to increase yield of wheat [10]. GWAS and biparental mapping are the two main approaches to identify loci associated with GSC and GPC. GWAS on a panel of 372 diverse European wheat varieties resulted in identification of marker trait association (MTA) on chromosome 6A (QGpc.ink-6A) which controls 23.42% variation of GPC and 13.20% of GSC, but with the opposite allelic effects [10]. GSC specific effects were found on chromosomes 2A, 2B, 3A, 3B, 4A, 6A, and 6B [10,17]. Chromosomal localization of some of these effects overlaps with loci involved in amylopectin (2A, 2B, 3A, 3B, 4A, 5A, 5B, 6A, 6B, 7A and 7B) and amylose synthesis (1B, 2A, 2B, 3A, 4A and 5A) active during the grain development process [9,17]. Mapping of quantitative trait loci (QTL) on different biparental populations revealed QTLs associated with GSCs on chromosomes 1A, 1B, 1D, 2A, 2D, 3B, 3D, 4A, 4D, 5A, 5B, 5D, 7A, 7B, and 7D [9,17,20,21]. Also, the waxy genes encoding granule-bound starch synthase (GBSSI, EC 2.4.1.21) are located at three loci Wx-A1, Wx-B1 and Wx-D1, on chromosomes 7AS, 4AL and 7DS, respectively [12,13,22,23,24]. GWAS revealed loci associated with the GPC on chromosomes 1A, 1B, 1D, 2A, 2B, 2D, 3A, 5A, 5B, 5D, 6A, 6B and 7B [9,10,25,26]. Some Single Nucleotide Polymorpism (SNP) markers useful for breeding to increase GPC have been selected [9,10,25]. QTL mapping approaches for GPC revealed the most important effects on chromosomes 6B and 7B. In addition, QTLs affecting GPC during grain development were detected on chromosomes 1B, 2A, 3B, 3D and 7A [9].

Wheat used in the heat-processed bakery products is a source of acrylamide. Acrylamide is formed from free asparagine and reducing sugars (glucose, fuctose, maltose) [27,28,29,30,31,32,33,34,35] i.e., in the Maillard reaction [31,36]. The free asparagine (Asn) is an amino acid that together with glutamate and glutamine in higher plants is responsible for the storage and transport of nitrogen [37,38]. The GFAC of grain products depends on the genotype [27,31,39] and increases under sulfur-deficient conditions [40,41]. GWAS resulted in the identification of MTAs for GFAC on chromosomes 1A, 1B, 2A, 2B, 2D, 3B, 4A, 4B, 5A, 6A, 6B, 6D, 7A, and 7B [39,42,43,44,45]. Aspartate synthase is the enzyme responsible for asparagine synthesis in plants. It catalyzes the ATP-dependent transfer of the amide group of glutamine to aspartate. The products of the reaction are glutamate and asparagine [37]. In common wheat, the asparaginase synthetase gene family consists of 5 genes per genome [38,46,47].

The purpose of the present study was to identify MTAs for GSC, GPC, and GFAC in common wheat elite breeding lines in GWAS. Cultivars with elevated protein contents and reduced levels of free asparagine may be more suitable for the bakery industry. The knowledge of regions responsible for different level of GSC, GPC, and GFAC in modern germplasms can be exploited to improve genetic gains and the breeding of varieties with desired set of traits.

## 2. Materials and Methods

### 2.1. Plant Material

In total, 343 elite breeding lines and 3 reference cultivars of common winter wheat (Table S1) from on-going breeding programs were planted at three research stations located in Poland at Kobierzyce (KBP, N 50°58′34″, E 16°55′53″), Smolice (SMH, N 51°41′58″, E 17°10′29″) and Strzelce (STH, N 52°18′52″, E 19°24′20″) in 2021–2022 and 2022–2023 cropping seasons. Therefore, 6 environments were assigned kob22, kob23, smh22, smh23, sth22 and sth23 for experimental stations located in Kobierzyce, Smolice and Strzelce, respectively, and cropping seasons 2022 and 2023. The experiments were set up in a split-block design in three sets of 59–60 genotypes including 3 standard cultivars (Artist, Formacja, and Kilimanjaro in 2022, and Artist, Formacja and Symetria in 2023) and 18 incomplete blocks per set. Each block consisted of 10 randomly assigned genotypes, accounting for three repetitions per genotype. Kernels were harvested from a 10 m^2^ plots (8 rows, 12.5 cm apart, and 10 m long), stored and used for chemical analyses.

### 2.2. Phenotypic Data Collection

Near infrared spectroscopy (NIR) (FOSS Infratec Nova analyzer, Hilleroed, Denmark) was used to determine the GSC, and GPC in the [%] unit. The mean humidity of grains was 11.4%. The samples collected in Strzelce in 2022 were used for determination of GFAC in flour. The isolation of free asparagine was carried out according to [48] with some modifications. The amount of wheat flour and acid added was reduced 4-fold from 10 g and 30 mL to 2.5 g and 7.5 mL, respectively. GFAC was determined on Varioskan LUX spectrophotometer (Thermo Fisher Scientific, Waltham, MA, USA) in 96-well plates according to the instructions provided with the K-ASAM L-ASPARAGINE/L-GLUTAMINE/AMMONIA (RAPID) reagent kit (Megazyme Bray, Co. Wicklow, Ireland). The volume of sample added was increased from 10 to 25 µL. Incubation times were increased from 5 min to 36 min, and 2 × 75 min between the first (conversion of L-glutamine into L-glutamate), the second (ammonia is converted to L-glutamate in the presence of NADPH, glutamate dehydrogenase (GIDH) and 2-oxoglutarate) and the third reaction (hydrolysis to L-aspartate and ammonium ions by asparaginase).

### 2.3. Genotyping

DNA was isolated from 2-week-old seedlings according to CTAB (cetyltrimethylammonium bromide) method recommended by Diversity Arrays Technology Pty Ltd. (Bruce, Australia). The quality of samples was checked on 1.5% agarose and DNA concentration was determined with spectrophotometer. In total, 343 winter wheat lines were genotyped with DArTseq markers in 2022 or 2023 season. Analysis of 170 genotypes carried out in 2022 resulted in 19,483 DArTseq markers (8952 silicoDArTs, and 10,531 SNPs). For the panel of 174 lines, 61,317 DArTseq markers (25,934 silicoDArTs and 38,933 SNPs) were obtained in 2023. Markers with minor allele frequencies higher than 0.05 and missing rate lower than 0.05 (on average 8% and 19% of missing data in 2022, and 2023, respectively) were used for the analysis.

### 2.4. Data Analysis

The distribution of the data, descriptive statistics, correlations and ANOVA was obtained with Statistica 13.3 software (Tibco, CA, USA). For the genotypes studied, the BLUP (Best Linear Unbiased Prediction) and the heritability were calculated using the R package—“Phenotype” [49]. It is based on the functionality of the lme4 package, which is used to fit linear models with mixed effects [50]. The heritability was calculated according to the formula: H^2^ = V_G_/(V_G_ + V_E_), where V_G_ and V_E_ denote the genetic and environmental variance components, respectively [51]. Markers spaced every 5 Mbp were selected for the analysis of the population structure [52]. Evenly distributed 2097 and 2782 SNP markers were obtained for sets of genotypes analyzed in 2022 and 2023, respectively. STRUCTURE v 2.2 [53] software was used to calculate the population structure with K values ranging from 1 to 15, iterated 10,000 times.

The BLUP values were subjected to GWAS. General Linear Model (GLM), Mixed Linear Model (MLM), and Compressed Mixed Linear Model (CMLM) models of the GAPIT package were tested with iPat (Intelligent Prediction and Association Tool) [54]. False Discovery Rates were calculated for the *p*-values to select significant effects.

### 2.5. Identification of Candidate Genes

Positions of selected MTA loci with the lowest *p*-values accompanied by the highest or main effect were established on IWGSC v2.1 genome sequence in the Unit Resources Genomics-Info database (URGI) [55] using the sequences of significant SNP markers. For selected MTAs with the highest effects, flanking genes located within 5 Mbp window were selected. Gene ontology (GO) annotations were checked on Ensembl Plants (http://plants.ensembl.org/ (accessed on 14 April 2025) and Uniprot (https://www.uniprot.org/ (accessed on 14 April 2025). The expression profiles of selected genes with functions directly or indirectly related to the traits studied were retrieved from WheatOmics 1.0 [56].

## 3. Results

### 3.1. Phenotypic Data

**Figure 1:**
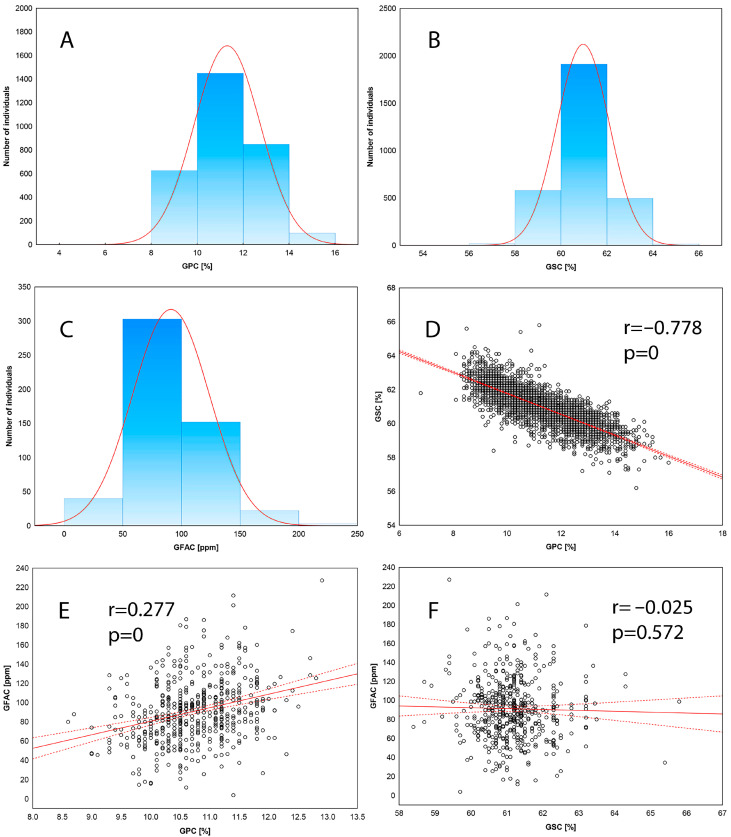
Distribution of grain protein content (**A**), grain starch content (**B**) and grain free asparagine content (**C**); correlations graphs between traits: GPC-GSC (**D**), GPC-GFAC (**E**), GSC-GFAC (**F**).

Analysis of variance showed significant variation in the average starch and protein contents in grains and a significant influence of environment for GSC and GPC. The average GSC in 344 lines of wheat was 60.97% and varied between environments in a narrow range from 60.16 (smh22) to 62.27% (sth23) (Table 1). Higher variation of GPC was found. On average, grains contained 11.31% protein and the content varied from 9.61% (sth23) to 12.92 (smh22). The greatest variability was found for GFAC, which ranged from 26.67 to 227.16 ppm in the varieties studied. With two exceptions, the absolute values of skewness and kurtosis were lower than 1.0. This indicates that the distribution of the studied traits is close to normal, and GSC, GPC and GFAC belong to the quantitative traits controlled by multiple loci (Figure 1). The highest broad-sense heritability reaching almost 80% were obtained for GPC, while for GSC we found 70% and 45% in panels of wheat lines analyzed in 2022 and 2023, respectively. A strong negative correlation between GSC and GPC (−0.778, *p* < 0.001) indicates, that both processes of proteins and starch accumulation during wheat grain maturation are linked, and selection for low GSC will result in an increase of GPC. A weak positive correlation (0.277, *p* < 0.001) was observed for the GPC-GFAC pair.

### 3.2. Genotype Variation

A total of 19,483 and 61,317 DArTseq markers were used for association analyses in 2022 and 2023, respectively. Some of these markers had an ambiguous position on the reference genome (IWGSC v.2.1), while other markers were mapped (Figure S1). The distribution of DArTseq markers on wheat chromosomes which is not random, and a distal fragment are apparently better saturated.

The coverage of the wheat genome with markers in 2023 was more than 3 times higher than in 2022 (Figure S1). Although DArTseq markers covered the entire wheat genome in both years, only 13,607 markers were common in both years of analysis and panels of common wheat lines (Figure S2).

### 3.3. Population Structure

**Figure 2:**
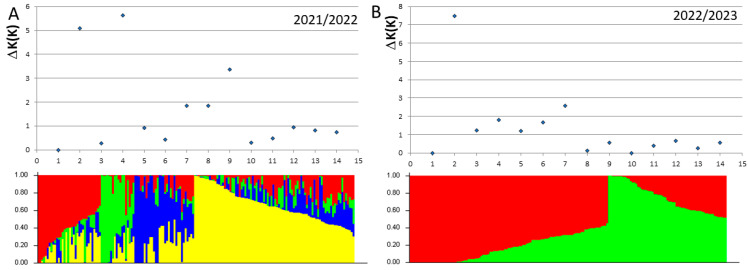
Number of subpopulations identified with 2079 (**A**) and 2782 (**B**) DArTseq markers in 170 and 174 elite breeding lines studied in 2021/22 and 2022/23 growth seasons, respectively. The colors denote the probability of a line being included in a given subpopulation.

Subsets of 2,097 and 2,782 of DArTseq markers were used for the population structure analysis of common wheat panels analyzed in 2022 and 2023, respectively. To avoid overrepresentation of genetic diversity deposited in distal regions of chromosomes, markers with the lowest number of missing data were selected from 5Mbp linkage blocks. Different genetic structure of elite breeding lines was found in subsequent years (Figure 2). In 2022 year, 170 lines were divided into four subpopulations represented by 86, 34, 32 and 18 lines. The panel of lines surveyed in 2023 consisted of two subpopulations represented by 111 and 63 lines (Table S1).

### 3.4. GWAS Results

**Figure 3:**
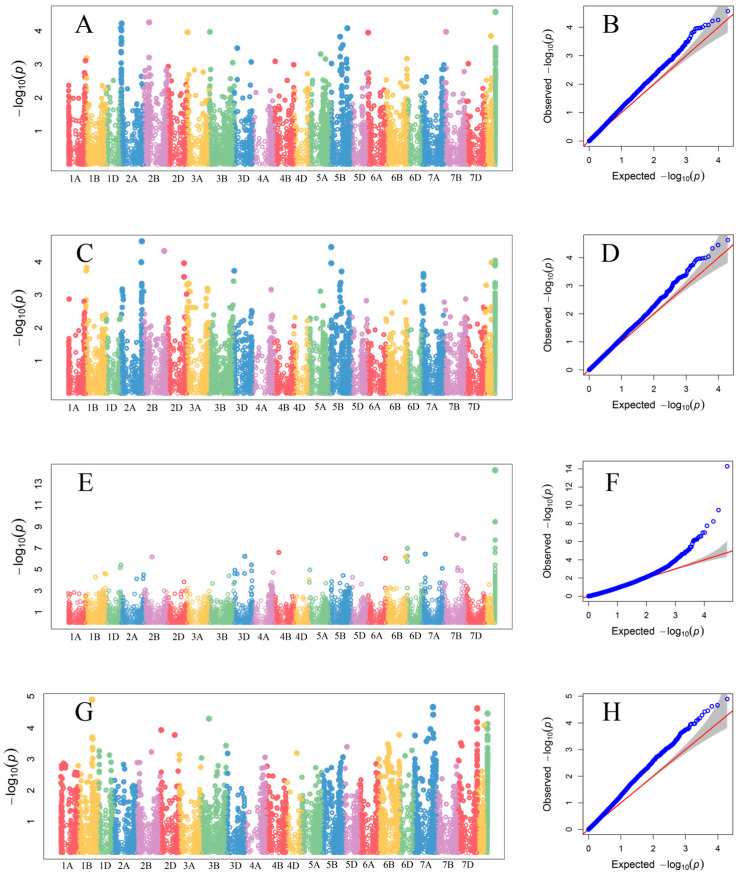
Manhattan and Q-Q plots obtained from GWAS (GLM) for: GPC 2022 (**A**,**B**), GSC 2022 (**C**,**D**), GSC 2023 (**E**,**F**), GFAC (**G**,**H**).

In total, 12 chromosomal regions associated with changes in protein content were found in the studied breeding lines using the GLM model (Figure 3). No major genes affecting GPC were identified and the markers explained from 6.8% to 10.2% of total variation (Table 2 and Table S2). The marker-trait association (MTA) effect of QGpc.rut.2D was stable for both panels of lines tested. Most MTAs were significantly associated with protein content only in the selected environments.

Genome-wide association studies for GSC revealed 40 MTAs (Table S3, Figure 3 and Figure S3) clustered in 35 QTLs, with DArTseq markers showing minor allele frequency (MAF) above 0.05. QGsc.rut.3D.4 was common for both panels of advanced breeding lines. Three models (GLM, MLM, and CMLM) produced consistent results for 16 MTAs (Table 3) identified for GSC measured in 2023 year. Selected markers explained from 6.8% to 12.2% (QGsc.rut.7A.4) of variation (Table S3).

In total, 43 markers associated with free asparagine content were identified on 15 chromosomes using the GLM model in 2022 (Figure 3, Table 4 and Table S4). These markers explained from 6.3% to 11.9% of the variation (QGfac.rut.1B.1). Distribution of MTAs was not random, and 6 markers have been located in the first homoeologous group on 1B in the fragment of 470–495 Mbp and on 1D in area of 368–423 Mbp. Similarly, the distribution of MTAs between chromosomes 2B and 2D was in the corresponding regions of 31 Mbp and 35 Mbp and 517 Mbp and 497 Mbp, respectively. Further, 3 MTAs have been located in the beginning of chromosomes belonging to the third 3 group. Two clusters of markers were also found. The first comprised 5 markers mapped in the 245–375 Mbp on chromosome 6B. The second group included 8 MTAs located on the long arm of chromosome 7A (Table 4).

### 3.5. Candidate Genes

Most of the markers associated with the traits studied were related to selected loci on the wheat genome, and it was not possible to distinguish narrow regions of the chromosome saturated with MTAs where candidate genes are located. The search for candidate genes was limited to regions adjacent to the selected markers, which included the nearest 5 genes upstream and downstream. For GPC, region adjacent to 4990459 marker (QGpc.rut.2D) which explained 10.2% of the variation and was stable between years was characterized, but no genes directly affecting GPC were identified (Table S5). For GSC, two markers 1280335 and 980786 from the region of QGsc.rut.7A.2, has been selected. These markers in spite of low frequency, overlapped with existing MTA (1127783). Sucrose synthase gene (TraesCS7A03G0375000) was identified in the region of QGsc.rut.7A.2 which is involved in the sucrose metabolism process. Finally, three MTAs with the GFAC were selected 4989859 (QGfac.rut.1B.1), 1081766 (QGfac.rut.3B.2), and 3953081 (QGfac.rut.7A.6), explaining 11.9%, 10.1%, and 11.2% of variation in GFAC, respectively. NAD(P)H dehydrogenase subunit H was identified near the region of QGfac.rut.1B.1 and can be indirectly related with nitrogen metabolism.

## 4. Discussion

The resulting locations of the 14 MTAs were compared with markers identified to date for GPC (Table S6). The cluster of 4 markers located in the 0.9–18.2 Mbp region on chromosome 2A (IWGSC v1.0) is a new region that does not overlap with previously described markers from this chromosome (located in the terminal region of the chromosome). Similarly, new GPC-related regions have been identified by the markers QGpc.rut.5B.1 (331 Mbp, v1.0), QGpc.rut.5B.2 (357 Mbp, v1.0), QGpc.rut.6A (4.7 Mbp, v1.0), 7B (66 Mbp, v1.0), and QGpc.rut.2D. The remaining markers QGpc.rut.3A, QGpc.rut.3B, QGpc.rut.5B.4 and QGpc.rut.5B.3 are located within a 50 Mbp window relative to the previously described markers (Table S6).

Most of the markers associated with GSC identified within this study have not been described before. New MTAs for GSC were found on chromosomes 1B, 2D, 3D, 5A, 5B, 7A and 7B. Markers QGsc.rut.4A.1 and QGsc.rut.4A.2 were located in the previously reported regions [17]. The locations of the other GSC-associated MTAs also do not match with the markers reported in the literature. Markers associated with variation in asparagine content in grains identified on chromosomes 2D (QGfac.rut.2D.1) and 7B (QGfac.rut.7B.9) were located on 13.7 Mbp and 0.4 Mbp, respectively, away from previously described markers [42]. Four markers (QGfac.rut.4A, QGfac.rut.7A.1, QGfac.rut.7D.1, and QGfac.rut.7D.2) were found within the 50 Mbp window in respect to the already reported regions (Table S6) [42,43].

The genes indirectly related to protein biosynthesis in wheat grain have been identified on chromosome 2D (Table S6). They encode enzymes which are involved in the plant’s response to the stress factor. Xyloglucan endotransglucosylases/hydrolases (XTH) are the key enzymes involved in cell wall remodeling, a process that includes relaxation and structural changes essential for the plant’s adaptation to stress [57]. The defense mechanisms may disturb the metabolic balance, including protein synthesis. The panel of diseases tolerance related genes in this region includes disease resistance protein RPM1 [58], NB-ARC domain-containing protein, disease resistance N-terminal domain-containing protein [59], and cytochrome P450 which modulates plant defense against Fusarium head blight [60]. Some of the enzymes in the P450 family also affect seed development and size [61].

**Figure 4:**
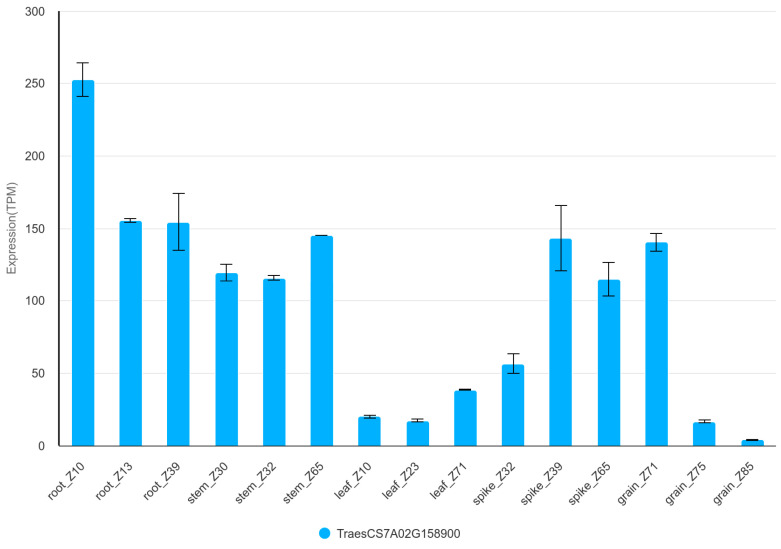
TraesCS7A02G158900 expression in organs during development of Chinese Spring plant. TPM—transcripts per million.

For GSCs, the protein directly involved in the starch biosynthetic pathway is sucrose synthase (SuS). This enzyme catalyses the reaction to convert sucrose into UDP-glucose and fructose [62], then UDP-glucose is converted to G1P [15]. The highest activity of this enzyme occurs at the time of increased starch synthesis and is mainly related to the endosperm development in *Triticum aestivum* [62]. The gene TraesCS7A03G0375000 (IWGSC v2.1) corresponds to TraesCS7A02G158900 (IWGSC v1.0) is located in the region of QGsc.rut.7A.2 set by markers 1280335 and 980786. This gene shows the highest level of expression in the root (>150 TPM), with slightly lower levels in the stem, spike and grain (Figure 4 and Figure S4). The lowest expression values are observed in the leaves (<50 TPM) [63]. In grain, the highest expression activity occurs early in grain development—10DPA (Figure S5) [64]. In the embryo, expression is higher than in the endosperm up to 14 DPA. After 25 DPA, it decreases significantly. In the endosperm, it remains at a similar level (Figure S6) [65].

**Figure 5:**
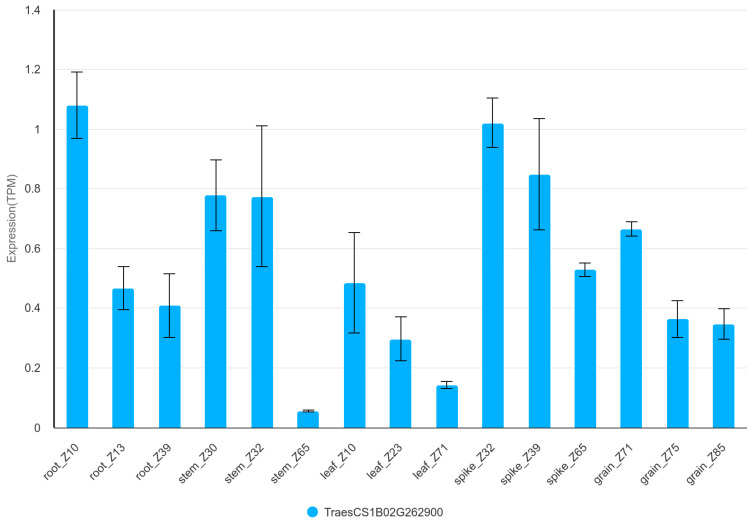
Expression of TraesCS1B02G262900 in organs during development of Chinese Spring plant. TPM—transcripts per million.

In the case of GFAC, gene TraesCS1B03G0736700 indirectly linked to the free asparagine content in the grain was identified on chromosome 1B. This gene coding NAD(P)H dehydrogenase is involved in the mitochondrial electron transport chain as the so-called complex I [66]. Metabolism of nitrogen is associated with this pathway as an element essential for the synthesis of amino acids and proteins [67]. Expression analysis of TraesCS1B03G0736700 (TraesCS1B02G262900 v1.1) shows that the gene is active in root, stem, leaf, spike and grain, respectively. Overall, the highest level of expression is observed in the spike (Figure 5 and Figure S7) [63]. Expression level of this gene is higher in the embryo (>1 TPM) than in the endosperm (<0.25 TPM) and remains at similar levels between 14 and 25 DPA (Figures S8 and S9) [65].

## 5. Conclusions

Association studies revealed 14 MTAs for GPC, 40 for GSC and 43 for GFAC, respectively. Polyploid nature of wheat and presence of homoeologous chromosomes introduce some level of uncertainty into physical mapping of markers to reference genome. However, beside several markers with overlapping position mainly new markers have been identified. In elite wheat breeding lines, main genes affecting important agronomically traits were possibly already fixed. Therefore only minor loci have been found that explained from 6.3% to 12.2% of phenotypic variation. The novel gene *TraesCS7A03G037500*, encoding sucrose synthase involved in starch biosynthesis, was identified in the proximity of QGsc.rut.7A.2 which opens further opportunity for better understanding of starch biosynthesis and accumulation processes. The reported findings provide valuable insights for elucidating inheritance of GCS. Identified MTAs provide molecular markers for reduction of free asparagine and an increase of protein content in wheat grains.

## Figures and Tables

**Table 1:** Phenotypic values for starch, proteins and asparagine content in grains of 344 winterwheat lines.

Trait	Mean± SD	Min	Max	Median	Skewness	Kurtosis	H^2^
GSC [%]	60.97 ± 1.14	56.2	65.8	61.0	0.01	0.32	
kbp22	60.48 ± 0.75	57.3	62.3	60.5	−0.56	0.93	0.70
smh22	60.16 ± 0.82	57.5	62.2	60.2	−0.15	−0.21	
sth22	61.15 ± 0.87	58.4	65.8	61.1	0.83	3.10	
kbp23	61.09 ± 0.82	57.9	63.3	61.1	−0.28	0.51	0.45
smh23	60.49 ± 1.24	56.2	64.2	60.5	0.00	0.13	
sth23	62.27 ± 0.72	60.0	65.6	62.2	0.46	0.73	
GPC [%]	11.31 ± 1.44	6.8	16.0	11.2	0.24	−0.55	
kbp22	12.37 ± 0.97	9.8	16	12.4	0.13	0.08	0.79
smh22	12.92 ± 0.99	10.4	15.7	12.9	0.04	−0.37	
sth22	10.73 ± 0.72	6.8	12.9	10.7	−0.24	1.46	
kbp23	10.92 ± 0.96	8.4	13.7	10.9	0.04	−0.36	0.78
smh23	11.71 ± 1.15	8.7	14.8	11.7	0.05	−0.38	
sth23	9.61 ± 0.65	8.10	11.70	9.60	0.36	0.11	
GFAC [ppm]	91.11 ± 32.71	26.67	227.16	89.79	0.52	0.97	0.51

**Table 2:** Significant marker-trait associations of DArTseq markers with BLUP values for GPC in elite breeding lines obtained for GLM. MAF—minor allele frequency.

Trait	MTA	DArTseq Marker	IWGSC v2.1	Position [Mbp]	*p*-Value	R2 [%]	MAF	Effect
GPC_2022	QGpc.rut.2A.1	1064413	2A	0.9	8.31 × 10^−5^	8.2	0.106	−0.673
GPC_2022	QGpc.rut.2A.2	1090321	2A	11.6	2.47 × 10^−4^	7.1	0.444	0.449
GPC_2022	QGpc.rut.2A.3	3961191	2A	18.2	9.73 × 10^−5^	8.0	0.424	0.572
GPC_2022	QGpc.rut.2A.3	7354314	2A	21.3	2.03 × 10^−4^	7.3	0.479	0.376
GPC_2022	QGpc.rut.2D	1090962	2D	16.6	1.43 × 10^−4^	7.6	0.344	0.281
GPC_2023	QGpc.rut.2D	4990459	2D	16.6	1.20 × 10^−5^	10.2	0.017	1.620
GPC_2022	QGpc.rut.3A	13880651	3A	10.5	1.09 × 10^−4^	7.9	0.088	−0.648
GPC_2022	QGpc.rut.3B	4004943	3B	23.2	1.07 × 10^−4^	7.9	0.309	0.297
GPC_2022	QGpc.rut.5B.1	16662440	5B	334.8	1.49 × 10^−4^	7.6	0.129	0.376
GPC_2022	QGpc.rut.5B.2	1058250	5B	359.8	3.30 × 10^−4^	6.8	0.109	0.390
GPC_2022	QGpc.rut.5B.3	3935268	5B	426.2	2.65 × 10^−4^	7.0	0.153	0.383
GPC_2022	QGpc.rut.5B.4	1385698	5B	534.3	3.36 × 10^−4^	6.8	0.076	0.493
GPC_2022	QGpc.rut.6A	1116192	6A	5.9	1.12 × 10^−4^	7.9	0.191	0.363
GPC_2022	QGpc.rut.7B	1080641	7B	68.7	1.06 × 10^−4^	8.0	0.068	0.594

**Table 3:** Significant marker-trait associations of DArTseq markers with BLUP values for GSC in elite breeding lines obtained for combination of GLM, MLM and CMLM.

MTA	DArTseq Marker	IWGSC v2.1	Position [Mbp]	*p*-Value	R2 [%]	MAF	Effect
QGsc.rut.1B.3	7352878	1B	644.8	2.43 × 10^−5^	10.9	0.230	−0.878
QGsc.rut.1B.4	5324459	1B	685.6	2.71 × 10^−5^	10.7	0.371	−0.390
QGsc.rut.3B	7353108	3B	105.1	2.09 × 10^−4^	8.3	0.236	−0.779
QGsc.rut.3D.2	1708238	3D	107.5	2.55 × 10^−5^	10.8	0.227	−0.829
QGsc.rut.3D.4	7353553	3D	613.1	2.21 × 10^−4^	8.2	0.164	−0.815
QGsc.rut.3D.4	7352096	3D	617.1	1.15 × 10^−4^	9.0	0.233	−0.777
QGsc.rut.4A.2	2256486	4A	695.4	2.64 × 10^−4^	8.0	0.417	−0.325
QGsc.rut.5A.1	1204378	5A	7.4	1.64 × 10^−4^	8.6	0.103	−0.519
QGsc.rut.5A.4	1059886	5A	569.7	1.93 × 10^−4^	8.4	0.086	−0.658
QGsc.rut.5B.4	1110565	5B	574.2	3.04 × 10^−4^	7.8	0.342	−0.322
QGsc.rut.6D	1066660	6D	477.9	2.59 × 10^−4^	8.0	0.187	0.753
QGsc.rut.7A.2	1127783	7A	116.1	1.57 × 10^−4^	8.6	0.057	−0.788
QGsc.rut.7A.4	4909952	7A	698.7	7.90 × 10^−6^	12.2	0.066	−0.912
QGsc.rut.7B.1	2276168	7B	8.6	3.08 × 10^−4^	7.8	0.374	0.288
QGsc.rut.7B.3	1067031	7B	470.7	1.25 × 10^−5^	11.7	0.052	−0.988
QGsc.rut.7B.4	3935071	7B	610.8	2.02 × 10^−4^	8.3	0.411	−0.311

**Table 4:** Significant marker-trait associations of DArTseq markers with BLUP values for GFAC in elite breeding lines obtained for GLM.

MTA	SNP Marker	IWGSC v2.1	Position [Mbp]	*p*-Value	R2 [%]	MAF	Effect
QGfac.rut.1B.1	4989859	1B	468.9	1.52 × 10^−5^	11.9	0.293	−14.28
QGfac.rut.1B.2	1023929	1B	481.0	1.53 × 10^−4^	8.5	0.207	13.48
QGfac.rut.1B.3	996356	1B	491.1	7.00 × 10^−4^	7.6	0.169	9.54
QGfac.rut.1B.3	1063426	1B	495.3	3.68 × 10^−4^	8.4	0.186	9.72
QGfac.rut.1D.1	985475	1D	20.5	3.39 × 10^−4^	7.3	0.494	7.89
QGfac.rut.1D.2	1043337	1D	368.0	1.07 × 10^−3^	6.9	0.130	14.40
QGfac.rut.1D.3	1128816	1D	423.0	1.06 × 10^−3^	6.3	0.112	11.33
QGfac.rut.2B.1	7940434	2B	31.5	1.06 × 10^−3^	6.3	0.337	7.09
QGfac.rut.2B.2	1021699	2B	111.5	1.01 × 10^−3^	6.3	0.157	10.43
QGfac.rut.2B.3	1201965	2B	516.7	9.11 × 10^−4^	7.2	0.210	9.03
QGfac.rut.2D.1	1019419	2D	35.8	1.32 × 10^−4^	9.1	0.154	−12.22
QGfac.rut.2D.2	2242065	2D	496.8	1.84 × 10^−4^	8.7	0.183	15.04
QGfac.rut.3A	1069217	3A	0.9	7.58 × 10^−4^	7.0	0.379	−7.16
QGfac.rut.3B.1	5005709	3B	2.5	9.50 × 10^−4^	6.7	0.388	−7.16
QGfac.rut.3B.2	1081766	3B	256.1	3.97 × 10^−5^	10.2	0.287	−14.40
QGfac.rut.3B.3	1101184	3B	837.2	2.92 × 10^−4^	7.8	0.402	8.07
QGfac.rut.3D	1109137	3D	40.6	6.99 × 10^−4^	7.1	0.183	−8.94
QGfac.rut.4A	983765	4A	698.2	4.96 × 10^−4^	6.7	0.266	−9.02
QGfac.rut.5B.1	3941721	5B	634.2	9.91 × 10^−4^	6.3	0.322	7.63
QGfac.rut.5B.2	1266853	5B	697.9	9.76 × 10^−4^	6.8	0.180	−8.50
QGfac.rut.5D	1139602	5D	122.2	4.82 × 10^−4^	7.7	0.414	−7.23
QGfac.rut.6B.1	1250105	6B	18.8	1.04 × 10^−3^	6.6	0.296	7.90
QGfac.rut.6B.2	4992737	6B	245.3	9.28 × 10^−4^	6.3	0.311	−7.54
QGfac.rut.6B.3	1009606	6B	313.1	8.62 × 10^−4^	6.7	0.479	−7.12
QGfac.rut.6B.4	1001121	6B	323.2	2.33 × 10^−4^	7.9	0.482	−8.28
QGfac.rut.6B.5	2322830	6B	356.0	3.10 × 10^−4^	7.6	0.476	−7.87
QGfac.rut.6B.6	3533239	6B	375.6	4.02 × 10^−4^	7.2	0.459	−7.42
QGfac.rut.6B.7	1089420	6B	708.3	2.51 × 10^−4^	8.7	0.376	8.32
QGfac.rut.6D.1	998928	6D	181.1	5.46 × 10^−4^	6.9	0.281	13.93
QGfac.rut.6D.2	1016778	6D	418.4	6.77 × 10^−4^	7.3	0.269	13.52
QGfac.rut.7A.1	1017632	7A	25.8	1.94 × 10^−4^	8.7	0.349	9.24
QGfac.rut.7A.2	1011371	7A	413.0	2.34 × 10^−4^	8.1	0.062	16.25
QGfac.rut.7A.3	1862702	7A	477.5	1.84 × 10^−4^	8.4	0.053	18.50
QGfac.rut.7A.4	994119	7A	549.9	8.87 × 10^−5^	9.2	0.068	16.99
QGfac.rut.7A.5	1696589	7A	622.0	5.22 × 10^−4^	7.1	0.139	14.74
QGfac.rut.7A.6	3953081	7A	638.9	1.86 × 10^−5^	11.2	0.189	12.71
QGfac.rut.7A.7	994476	7A	656.7	1.71 × 10^−4^	8.4	0.062	17.59
QGfac.rut.7A.8	1331106	7A	702.8	9.12 × 10^−4^	7.1	0.322	8.10
QGfac.rut.7B.9	985944	7B	756.0	8.64 × 10^−4^	6.5	0.210	8.56
QGfac.rut.7D.1	2269456	7D	15.4	8.07 × 10^−4^	6.5	0.080	18.25
QGfac.rut.7D.2	1022222	7D	77.7	1.97 × 10^−4^	8.0	0.464	10.81
QGfac.rut.7D.3	1062859	7D	114.5	2.91 × 10^−4^	7.8	0.192	10.88
QGfac.rut.7D.4	1236791	7D	633.0	5.49 × 10^−5^	9.9	0.115	14.02

## Data Availability

The data are contained within the article or Supplementary Material.

## Supplementary Materials

The following supporting information can be downloaded at: https://www.mdpi.com/article/10.3390/genes16060661/s1, Figure S1: Physical distribution of 14,345 (A) and 46,586 (B) DArTseq markers on wheat chromosomes (IWGSC v.2.1) used in association mapping in 2021/22 and 2022/23, Figure S2: Comparison of DArTseq marker sets used in association analyses in 2022 and 2023, Figure S3: Manhattan and qq-plots obtained from GWAS for: GSC 2022—MLM (A,B), GSC 2022—CMLM (C,D), GSC 2023—MLM (E,F), GSC 2023—CMLM (G,H), Figure S4:TraesCS7A02G158900 expression in tissues of Chinese Spring; Figure S5: TraesCS7A02G158900 expression in developing wheat grain; Figure S6: Expression of TraesCS7A02G158900 in the embryo and endosperm of developing grain; Figure S7: TraesCS1B02G262900 expression in organs of Chinese Spring; Figure S8: Expression of TraesCS1B02G262900 in the embryo and endosperm of developing grain; Figure S9: TraesCS1B02G262900 expression in developing wheat grain; Table S1: Accessions used in the study, pedigree, subpopulation and BLUP values; Table S2: Markers associated with GPC in elite breeding lines. Alternative genomic localizations and sequences. Kbp, smh, and sth correspond to Kobierzyce, Smolice and Strzelce experimental stations; Table S3: Markers associated with GSC in elite breeding lines. Alternative genomic localizations and sequences. Kbp, smh, and sth correspond to Kobierzyce, Smolice and Strzelce experimental stations; Table S4: Markers associated with GFAC in elite breeding lines. Alternative genomic localizations and sequences; Table S5: Genes identified in the proximity of selected MTAs, and predicted functions; Table S6: MTAs and QTLs identified for GSC, GPC and GFAC. References [68] are cited in the supplementary materials.

## Abbreviations

ADP-ase	ADP-glucose pyrophosphorylase
ADPG	ADPG—ADP-glucose
AMP	adenosine monophosphate
Asn	asparagine
ATP	adenosine triphosphate
DBE	starch debranching enzymes
FK	fructokinase
G1P	glucose-1-phosphate
G6P	glucose-6-phosphate
GBSS	granule-bound starch synthase,
GPC	grain protein content
GSC	grain starch content
HK	hexokinase
INV	invertase
ISA	isoamylase-type starch debranching enzyme
NIR	near infrared spectroscopy
PGI	phosphoglucose isomerase
PGM	phosphoglucomutase
PPi	pyrophosphate
PUL	pullanase
QTL	quantitative trait loci
SBE	starch branching enzyme
SS	starch synthase
SuSy	sucrose synthase
UDPG	UDP-glucose
