# Supplementary material for: Markers Associated with Starch, Protein and Asparagine Content in Grain of Common Wheat
Source: Genes (Basel). 2025 May 29;16(6):661. doi: 10.3390/genes16060661 (PMC12193179; doi:10.3390/genes16060661)
Supplement: Supplementary file 1 [file genes-16-00661-s001.zip › genes-3616078-Figure S1-S9.pdf]

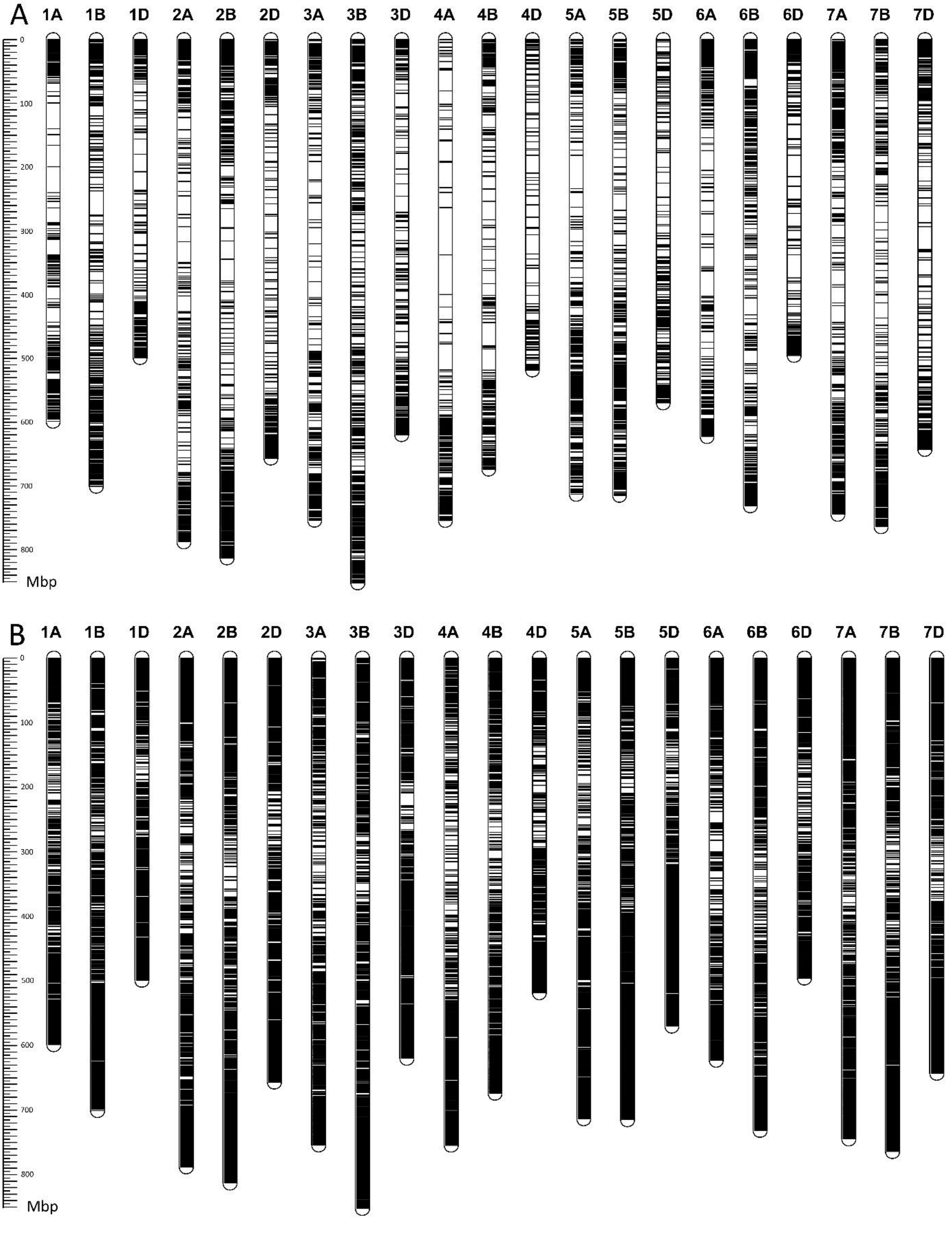

Figure S1. Physical distribution of 14,345 (A) and 46,586 (B) DArTseq markers on wheat chromosomes (IWGSC v.2.1) used in association mapping in 2021/22 and 2022/23.

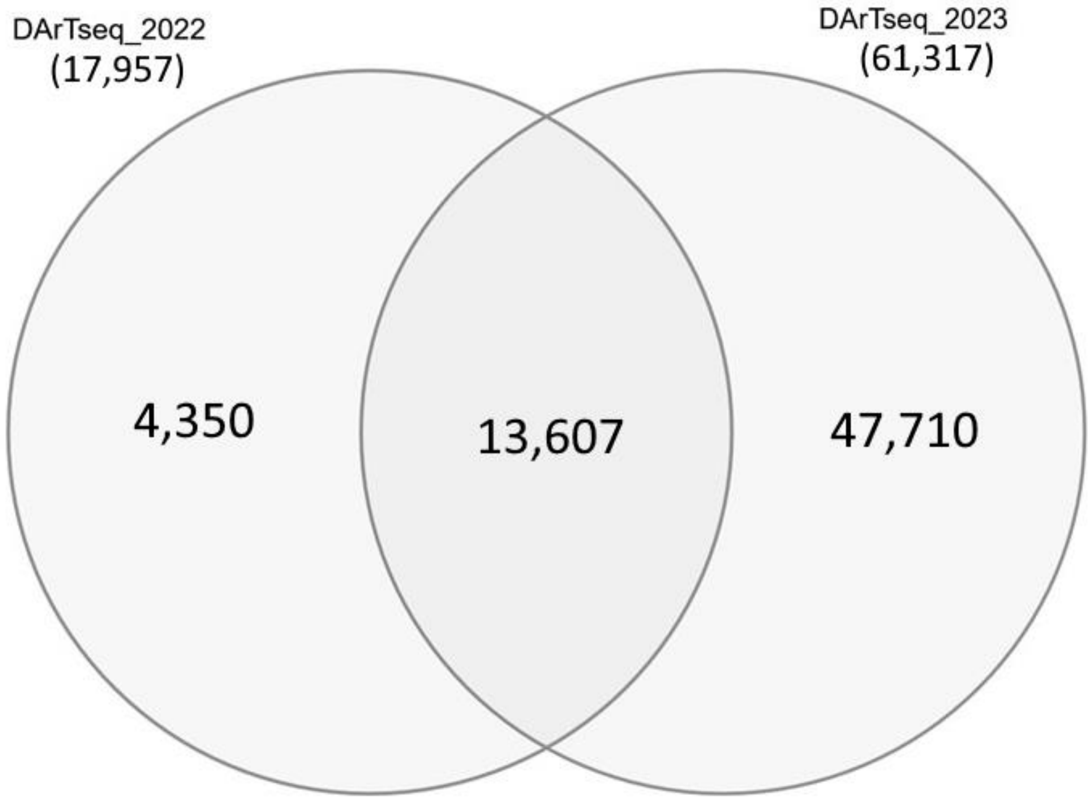

Figure S2. Comparison of DArTseq marker sets used in association analyses in 2022 and 2023.

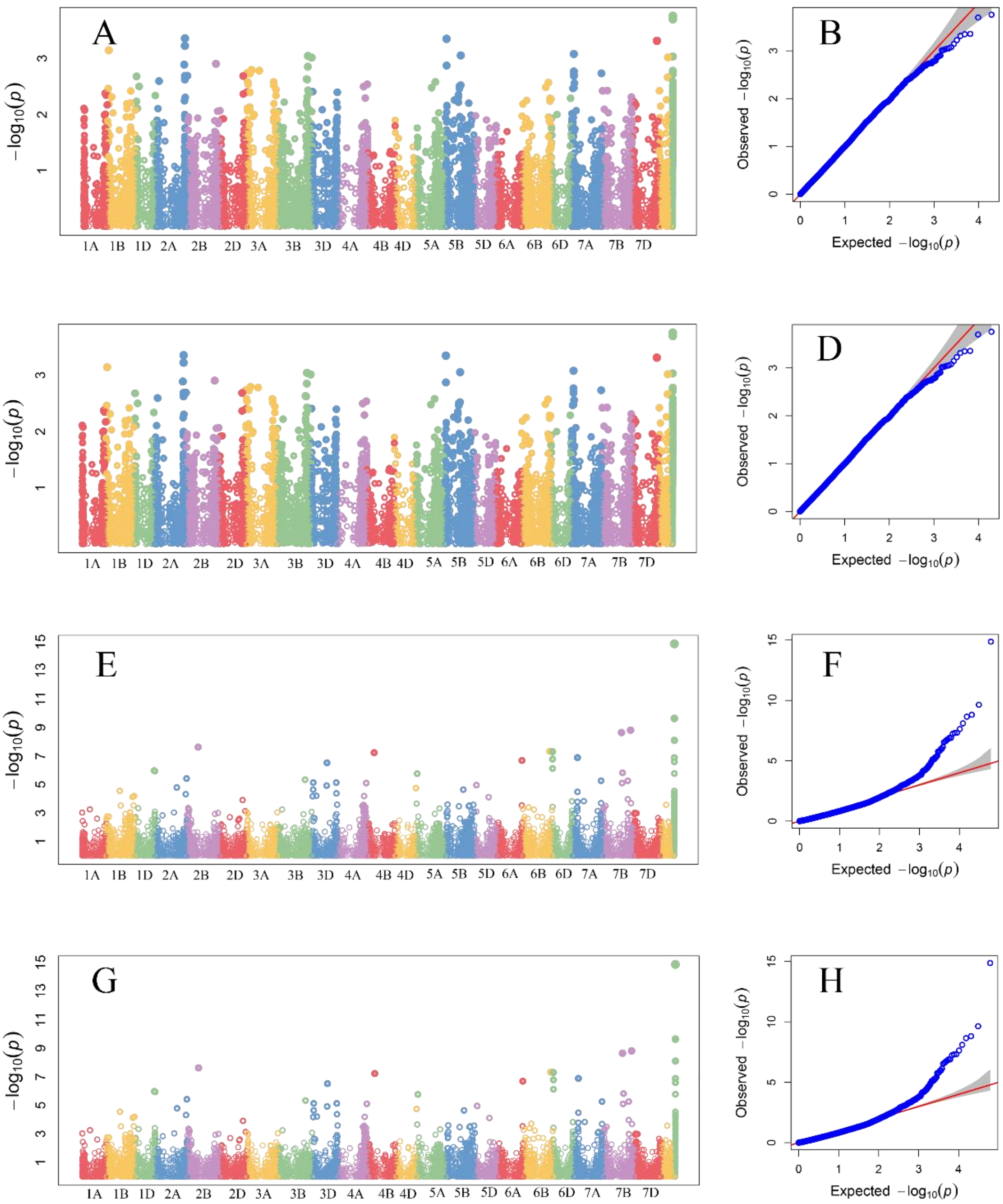

Figure S3. Manhattan and qq-plots obtained from GWAS for: GSC 2022 - MLM (**A, B**), GSC 2022 - CMLM (**C, D**), GSC 2023 - MLM (**E, F**), GSC 2023 - CMLM (**G, H**).

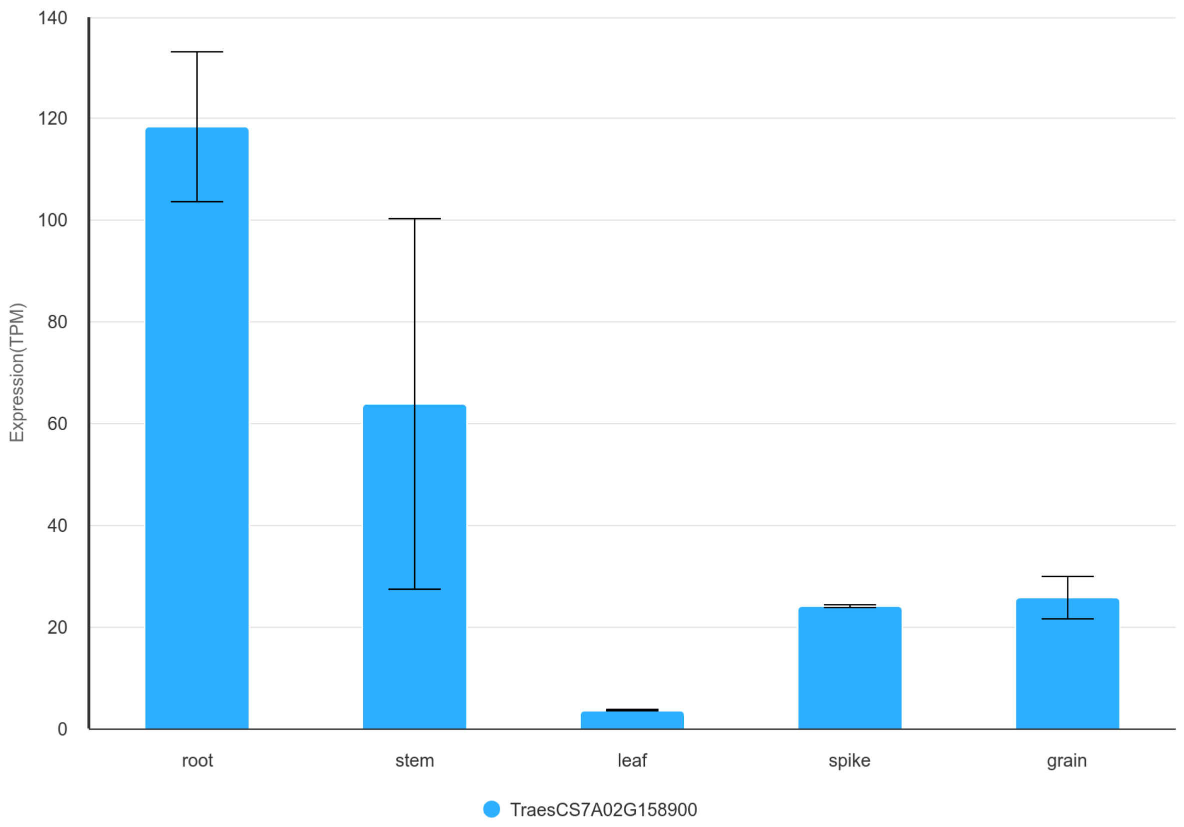

Figure S4. *TraesCS7A02G158900* expression in tissues of Chinese Spring [1,2].

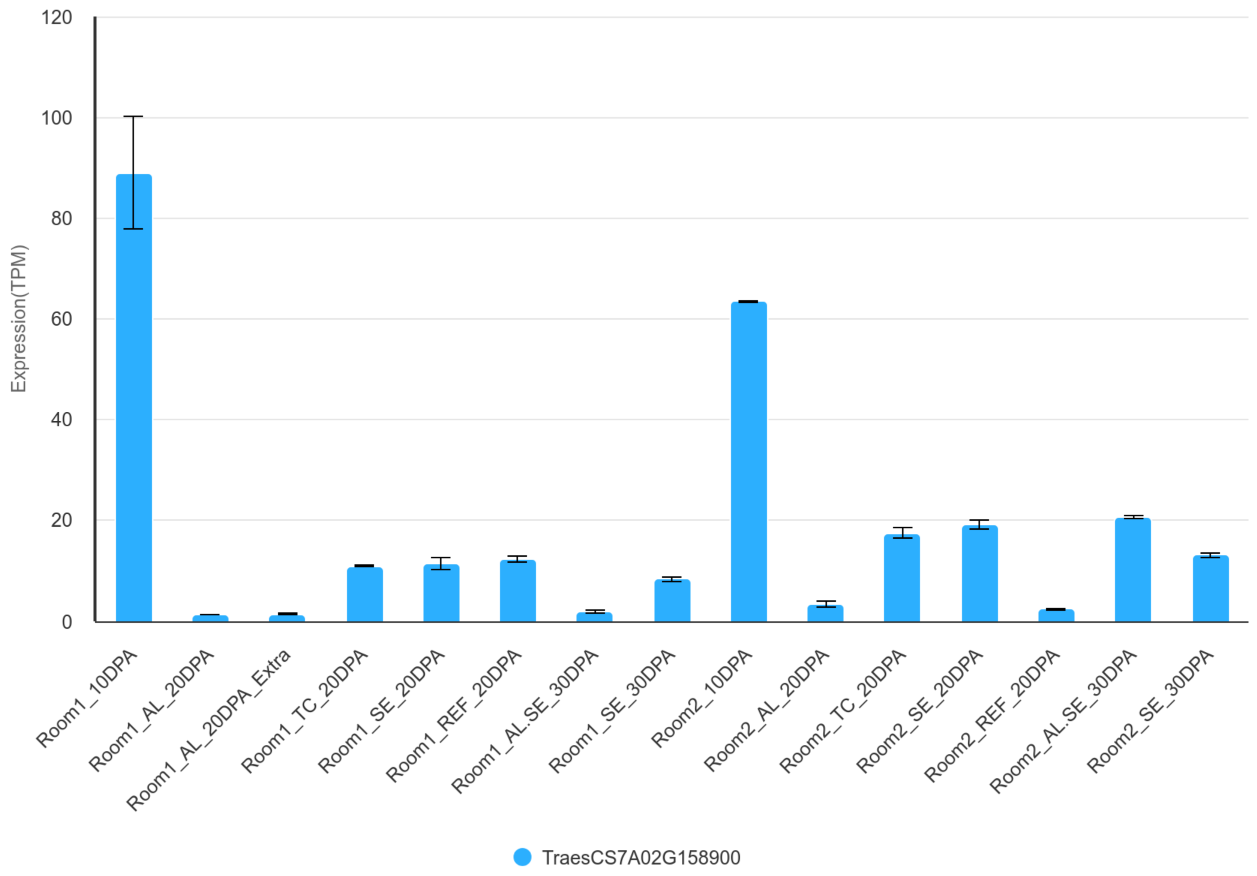

Figure S5. *TraesCS7A02G158900* expression in developing wheat grain (v1.1) [2,3]. DPA – days post anthesis.

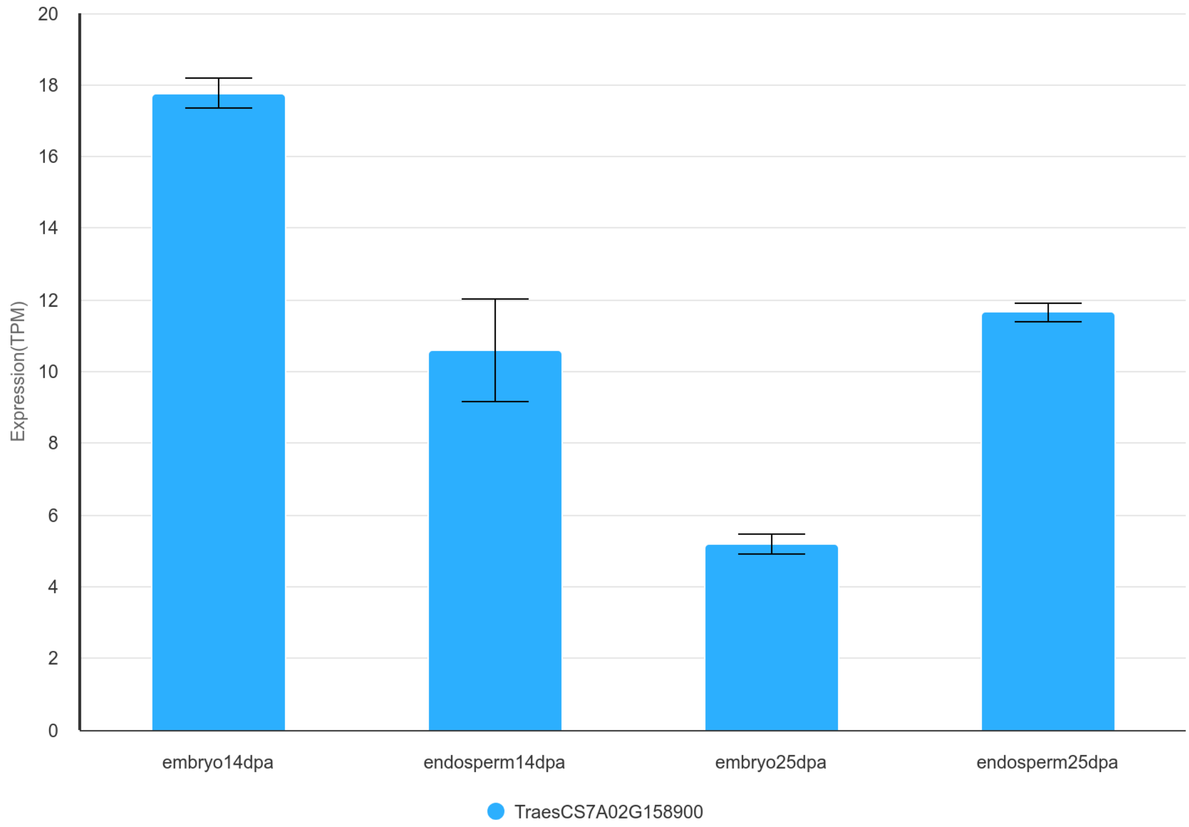

Figure S6. Expression of *TraesCS7A02G158900* in embryo and endosperm of developing grain [2,4].

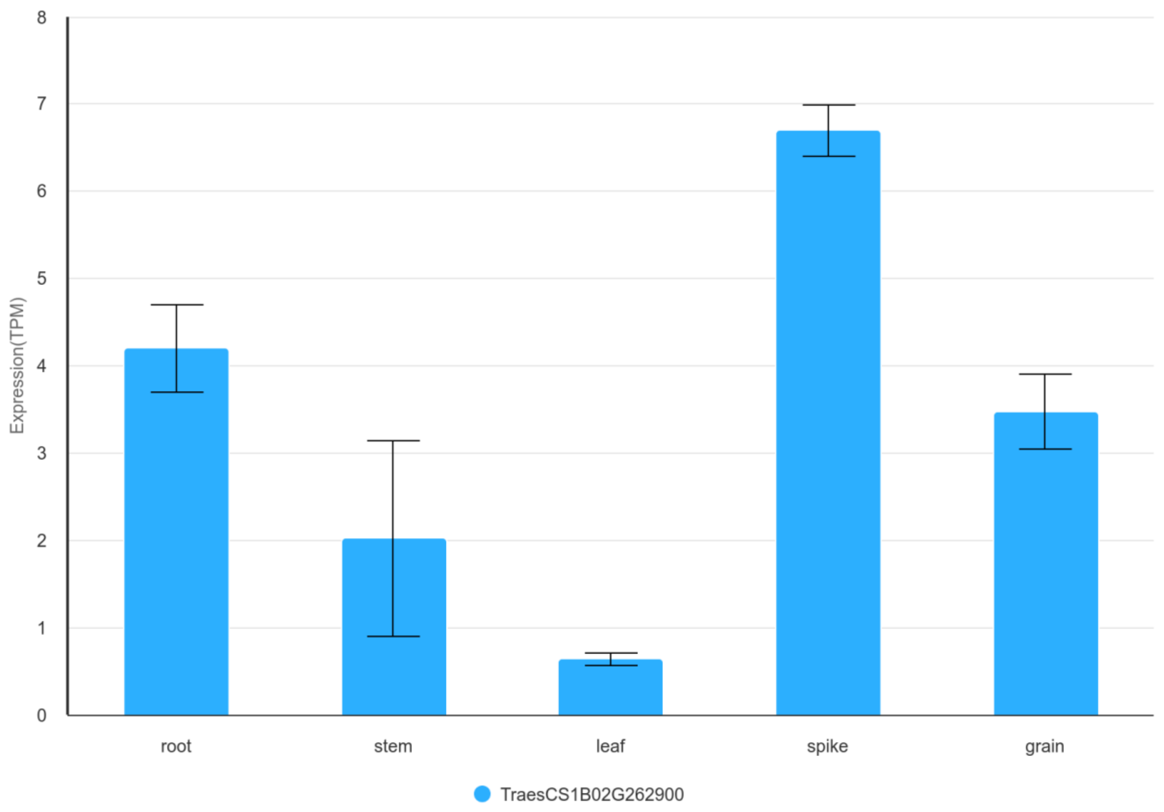

Figure S7. *TraesCS1B02G262900* expression in organs of Chinese Spring [1,2].

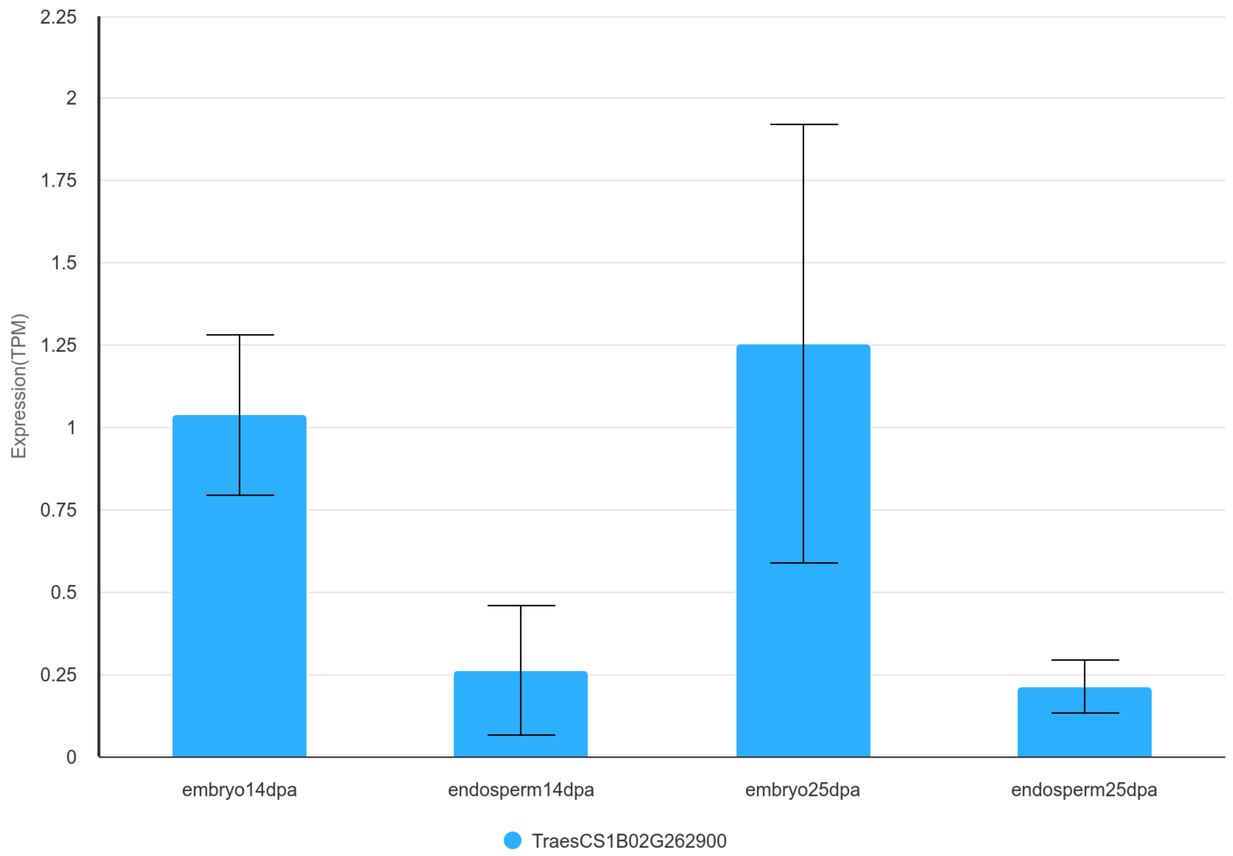

Figure S8. Expression of *TraesCS1B02G262900* in embryo and endosperm of developing grain [2,4].

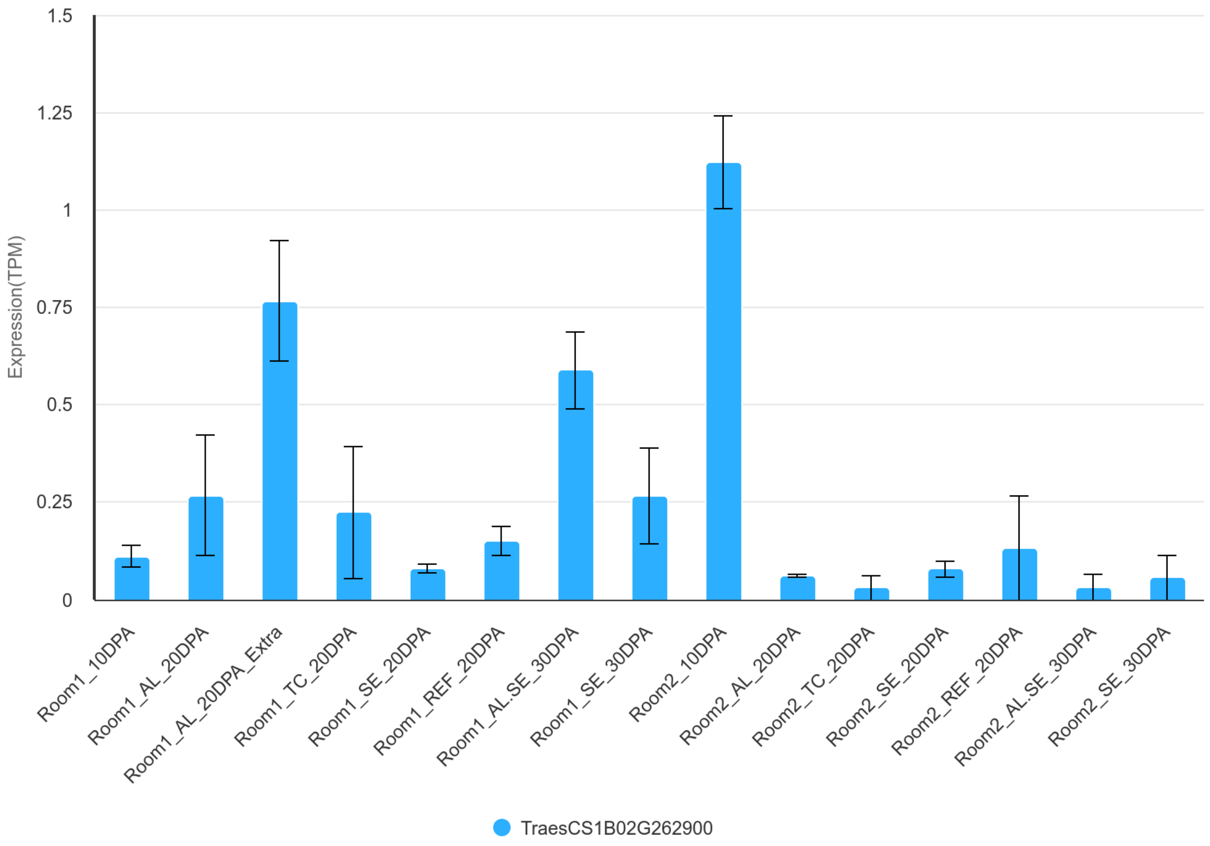

Figure S9. *TraesCS1B02G262900* expression in developing wheat grain [2,3].

1. Lukaszewski, A.J.; Alberti, A.; Sharpe, A.; Kilian, A.; Stanca, A.M.; Keller, B.; Clavijo, B.J.; Friebe, B.; Gill, B.; Wulff, B.; et al. A Chromosome-Based Draft Sequence of the Hexaploid Bread Wheat (Triticum Aestivum) Genome. *Science (1979)* **2014**, *345*, 2025, doi:10.1126/SCIENCE.1251788/SUPPL_FILE/MAYER.SM.PDF.
2. Ma, S.; Wang, M.; Wu, J.; Guo, W.; Chen, Y.; Li, G.; Wang, Y.; Shi, W.; Xia, G.; Fu, D.; et al. WheatOmics: A Platform Combining Multiple Omics Data to Accelerate Functional Genomics Studies in Wheat. *Mol Plant* **2021**, *14*, 1965–1968, doi:10.1016/J.MOLP.2021.10.006.
3. Pfeifer, M.; Kugler, K.G.; Sandve, S.R.; Zhan, B.; Rudi, H.; Hvidsten, T.R.; Mayer, K.F.X.; Olsen, O.A.; Rogers, J.; Doležel, J.; et al. Genome Interplay in the Grain Transcriptome of Hexaploid Bread Wheat. *Science (1979)* **2014**, *345*, 2025, doi:10.1126/SCIENCE.1250091/SUPPL_FILE/PFEIFER-SM.PDF.
4. Wei, J.; Cao, H.; Liu, J. dong; Zuo, J. hong; Fang, Y.; Lin, C.T.; Sun, R. ze; Li, W. long; Liu, Y. xiu Insights into Transcriptional Characteristics and Homoeolog Expression Bias of Embryo and De-Embryonated Kernels in Developing Grain through RNA-Seq and Iso-Seq. *Funct Integr Genomics* **2019**, *19*, 919–932, doi:10.1007/S10142-019-00693-0/FIGURES/8.
